# Efficacy of local anaesthetic and steroid combination in prevention of post-herpetic neuralgia: A meta-analysis

**DOI:** 10.12669/pjms.38.3.5140

**Published:** 2022

**Authors:** Xiaolu Zhang, Zhiwei Wang, Yiyuan Xian

**Affiliations:** 1 Xiaolu Zhang M.D, Department of Anesthesiology, The Fifth People’s Hospital of Chongqing; No. 24, Renji Road, Chongqing, 400062, P.R. China; 2 Zhiwei Wang M.D, Department of Anesthesiology, The Fifth People’s Hospital of Chongqing; No. 24, Renji Road, Chongqing, 400062, P.R. China; 3 Yiyuan Xian M.D, Department of Anesthesiology, The Fifth People’s Hospital of Chongqing; No. 24, Renji Road, Chongqing, 400062, P.R. China

**Keywords:** Post-herpetic neuralgia, Herpes zoster rash, Anesthesia, Steroid, Management, Biological mechanism

## Abstract

**Objective:**

The objective was to provide synthesized evidence on the efficacy of local anaesthetics and steroid injections for prevention and management of PHN, compared to the standard treatment using anti-viral and analgesic medications. The primary outcomes of interest were incidence of PHN and duration of neuralgic pain.

**Methods:**

Comprehensive searches were done systematically through PubMed, Scopus, Cochrane Central Register of Controlled Trials and Google scholar databases. Randomized controlled trials that compared the efficacy of local anaesthetics and steroid injections for preventing and managing PHN were included for this meta-analysis. A comprehensive search was done for papers published until 15^th^ July 2021.

**Results:**

A total of 10 RCTs were included in the meta-analysis. In the overall pooled analyses, compared to standard care/placebo, those receiving a combination of local anaesthetic and steroid injection had 55% lower risk of PHN at 3 months from onset of rash (RR 0.45; 95% CI, 0.29; 0.70). Out of the different modes of intervention delivery i.e., intravenous, subcutaneous and nerve block, maximum beneficial effect in reducing the incidence of PHN was noted in nerve block (RR 0.55; 95% CI, 0.34, 0.89).

**Conclusions:**

The meta-analysis provides some evidence to support the use of combined local anaesthetic and steroids in reducing risk of post-herpetic neuralgia and duration of neuralgic pain in patients with herpes zoster rash.

## INTRODUCTION

Reactivation of the varicella zoster virus in the dorsal root ganglion, post the primary infection, leads to Herpes Zoster.^1^ The condition is primarily encountered when the cellular immunity is compromised. Usual clinical presentation in the acute phase is of dermatomal vesicular rash, which is unilateral.^1^,^2^ One of the common debilitating complications is the Post-herpetic neuralgia (PHN) and is clinically considered to be present when, after the onset of rash, the Herpes Zoster associated pain continues for more than 90 days.^3^,^4^ Epidemiological data indicates that the incidence of PNH varies from 5% to more than 30% depending upon the operational definitions used, characteristics of the population studies and the study methodology adopted.^2^–^4^ Further, studies suggest that the intensity of the pain is influenced by age as well as presence of other risk factors such as prodromal pain, severity of pain during acute episode, severity of rash, immunological state and presence or absence of diabetes.^3^,^4^

It is important to understand the key biological mechanism that leads to PHN so that effective treatment strategies could be developed. Herpes zoster is often associated with inflammation of the dorsal root ganglion and the associated peripheral nerves and this inflammation is widely implicated as the causative factor for PHN.^4^,^5^ Therefore, it would be logical to consider treatment strategies that aim to reduce inflammation and thereby, alleviate pain. Existing studies have evaluated the role of local anaesthetics and steroids in the treatment and management of PHN. In a meta-analysis by Kim et al published in 2017, efficacy of nerve block using local anaesthetics and/or steroid injections have been reviewed.^6^ The authors found that the incidence of PHN in the group receiving nerve block reduced by 57%, in comparison to the standard of care group. Also, application of nerve block reduced the duration of zoster related pain. While this review is informative, there is a need to update this review as new trials have been published and their inclusion in the meta-analysis will be pertinent.

The purpose of this meta-analysis is to provide the most updated estimates on the efficacy of local anaesthetics and steroid injections for preventing and managing PHN, compared to the standard treatment using anti-viral and analgesic medications. The primary objective was to evaluate incidence of post-herpetic neuralgia at three months from start of rash and the alteration in the duration of pain with use of a combination of local anaesthetics and corticosteroids injections against standard medical treatment or placebo. We considered the incidence of PHN at 3 months from start of rash as PHN is clinically considered to be present when, after the onset of rash, the Herpes Zoster associated pain continues for more than 90 days.

## METHODS

A comprehensive search was done systematically through PubMed, Scopus, CENTRAL (Cochrane Central Register of Controlled Trials) and Google scholar databases for papers published until 15^th^ July, 2021. Free text words and medical subject heading (MeSH) terms were used. The key aim was to identify randomized controlled trials that evaluated the effect of local anaesthetics and steroid injections for preventing and managing PHN, compared to the standard treatment using anti-viral and analgesic medications on incidence of post-herpetic neuralgia and duration of pain. Studies that reported relevant outcome measures of interest to this meta-analysis were potentially considered for inclusion.

Two authors reviewed citations and selected studies. After removing the duplicates, screening of titles and abstracts was performed as a first step. Thereafter, review of the full text of potential studies was done. Any discrepancies related to the inclusion of studies were resolved through detailed discussion among the study authors. Only those studies were selected for the meta-analysis that adequately suited the inclusion criteria. The bibliographic list of the identified studies and relevant reviews on the subject were examined for additional possible studies.

### Inclusion Criteria

For a study to be included in the meta-analysis, it should have been a randomized controlled trial or a quasi-experimental trial conducted among subjects experiencing Herpes Zoster within 14 days after onset of the rash. The study should have compared the efficacy of local anaesthetics and steroid injections for preventing and managing PHN, compared to the standard treatment using anti-viral and analgesic medications/placebo.

### Exclusion Criteria

Non-randomized studies such as case-reports, observational studies (case-control, cohort studies) or review articles were excluded. Also, those studies that did not compare a “combination” of local anaesthetic and corticosteroid injections against “standard care/placebo” were excluded.

Extraction of relevant data from included studies was done by two authors independently, using a data extraction sheet. Following data from eligible studies were extracted: surname of first author, year in which the study was published, geographical location where the study was done, design of the study, characteristics of the study subjects, study groups and key findings of the study. The methodological assessment was done independently by two authors using the assessment tool by Cochrane.^7^

### Statistical Analysis

Statistical analysis was done using STATA version 13.0. Effect sizes were reported as weighted mean differences (WMD) for continuous outcomes. For categorical outcomes, pooled relative risks were reported. All estimates were reported with 95% confidence intervals (CI). Subgroup analysis was done based on the mode of administration of the local anaesthetic and steroids i.e., intravenous, subcutaneous or nerve block. Nerve block meant injecting the local anaesthetic and steroid in a localized area to block spinal nerves and included epidural, paravertebral and stellate ganglion block. Heterogeneity of effects was assessed and quantified by the I^2^. I^2^ value >50% was considered to represent substantial heterogeneity 8. In cases with substantial heterogeneity, random effects model was used.^8^ A p-value of <0.05 was considered statistically significant. Publication bias was assessed using Egger’s test and visually inspected using funnel plots. Quality of the evidence generated was assessed using GRADE criteria and categorized as “High”, “Moderate”, “Low” or “Very low”.^9^

## RESULTS

**Fig.1:**
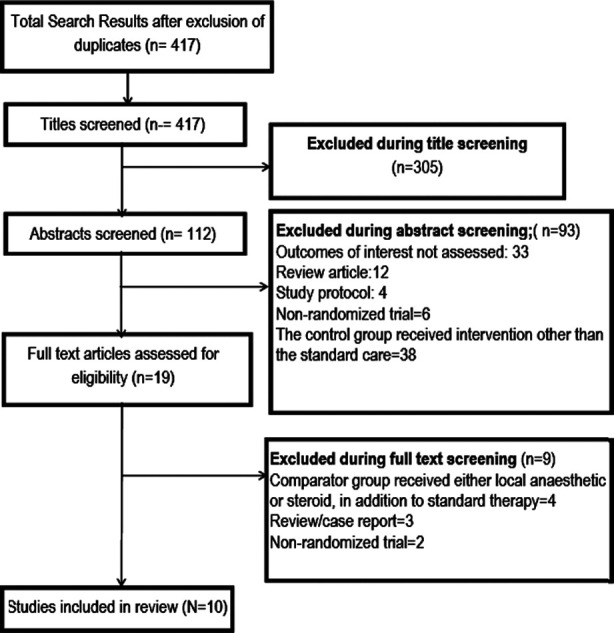
Selection process of the studies included in the review.

A total of 417 unique citations were obtained upon executing the search strategy in the PubMed, Scopus, CENTRAL (Cochrane Central Register of Controlled Trials) and Google scholar databases (Figure 1). Out of these, 305 were excluded based on title screening. Further, 93 citations were excluded after reading of the abstract. Full text of the remaining 19 articles were reviewed. Out of these, nine articles were excluded upon full text review. The final number of included articles in this meta-analysis was 10.^10^–^19^ Supplementary Table-1 presents the key characteristics of the included studies along with the key findings. All the included studies were randomized clinical trials, except one, which was a prospective non-randomized comparative clinical trial 17. Five studies were done in China, two studies each in Egypt and South Korea and one study in Netherlands. All the studies, except one, adopted random sequence generation; allocation concealment, blinding of participants and blinding of study personnel was adequately reported in eight out of 10 studies; in nine studies, blinding of the outcome assessment team was reported to be done. Overall, the included studies had good quality.

**Fig.2:**
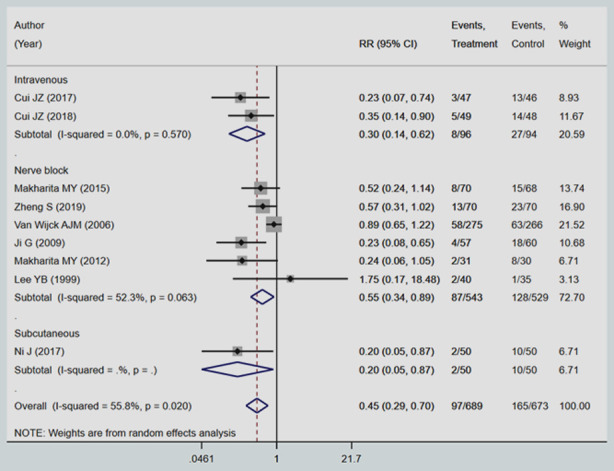
Pooled effect of local anaesthetics and steroid injections on incidence of post-herpetic neuralgia at 3 months from onset of rash.

The analysis based on the route of administration showed that the maximum beneficial effect was noted in nerve block (RR 0.55; 95% CI, 0.34, 0.89; I^2^=52.3%) followed by intravenous route (RR 0.30; 95% CI, 0.14, 0.62; I^2^=0.0%) (Fig.2). Only one study reported evidence on subcutaneous route and the effect size was significant, although with wide confidence intervals (RR 0.20; 95% CI, 0.05, 0.87).

**Supplementary Table-I:** Key details of the studies included in the meta-analysis.

Author, year of publication	Country	Study design	Subjects	Intervention and control groups	Key outcome
Cui JZ (2017)^10^	China	RCT	Ages 50–80 years old, experiencing <break/>Herpes Zoster within 7 days after onset of the rash and HZ pain intensity ≥4 on the visual analog scale (VAS).	Control: Antiviral medication along with analgesics Intervention: Antiviral medication along with analgesics AND repeated intracutaneous injections of 15 ml 0.25% ropivacaine and 40mg methylprednisolone every 48 h for a week (total 4 injections)	Incidence of Post-herpetic neuralgia (PHN) at 12 weeks Control: 13/46 (28.3%); Intervention: 3/47 (6.4%) Mean (SD) duration of pain (in days) Control: 58.2 (66.5); Intervention: 25.7 (37.2) Mean (SD) VAS score at 12 weeks Control: 1.7 (2.9); Intervention: 0.49 (1.9) Adverse effects Nausea/vomiting Control: 4/46 (8.7%); Intervention: 5/47 (8.5%) Drowsiness Control: 3/46 (6.5%); Intervention: 3/47 (6.1%)
Cui JZ (2018)^11^	China	RCT	Ages 50–80 years old, experiencing Herpes Zoster within 7 days after onset of the rash and HZ pain intensity ≥4 on the visual analog scale (VAS).	All patients received standard treatment (800 mg acyclovir 5 times daily and 150 mg pregabalin twice daily for 7 days) Intervention: Intra-cutaneous injection of 15 mL of 40 mg methylprednisolone plus 14 mL of 0.25% ropivacaine Control: intra-cutaneous injection of 15mL normal saline	Incidence of Post-herpetic neuralgia (PHN) at 12 weeks Control: 14/48 (29.2%); Intervention: 5/49 (10.2%) Mean (SD) duration of pain (in days) Control: 59.2 (65.0); Intervention: 28.4 (46.7) Mean (SD) VAS score at 12 weeks Control: 1.3 (2.2); Intervention: 0.6 (1.7) Adverse effects Drowsiness in the first week of treatment Control: 22/48 (45.8%); Intervention: 21/49 (42.6%)
Makharita MY et al (2015)^12^	Egypt	RCT	Patients over 50 years who had chest wall herpetic eruption of less than 1 week with moderate and severe pain	All patients received pregabalin in a dose of 150 mg twice daily. Control: Nerve block using 10 mL saline as placebo under fluoroscopy Intervention: Nerve block with 25 mg bupivacaine 0.5%, plus 8 mg dexamethasone in a total volume of 10 mL under fluoroscopy	Incidence of Post-herpetic neuralgia (PHN) at 12 weeks Control: 15/68 (22.1%); Intervention: 8/70 (11.4%) Mean (SD) duration of pain (in days) Control: 35.9 (29.1); Intervention: 24.6 (23.7) Mean (SD) VAS score at 12 weeks Control: 0.69 (1.36); Intervention: 0.40 (1.13) Adverse effects Drowsiness in the first week of treatment Control: 32/68 (47.1%); Intervention: 29/70 (41.4%)
Ni J et al (2017)^13^	China	RCT	Age ≥ 50 years; Herpes Zoster infection (with rash duration <7 days) and pain numerical rating scale (NRS) score >3	All patients received standard treatment i.e. analgesics (as needed) and antiviral medication (oral acyclovir 800 mg 5 times daily) Intervention: In addition to the standard treatment received a combination of triamcinolone (10 mg) and lidocaine (0.5%), injected subcutaneously	Incidence of Post-herpetic neuralgia (PHN) at 12 weeks Control: 10/50 (20.0%); Intervention: 2/50 (4.0%) No serious adverse events were noted in any of the two groups
Zheng S et al (2019)^14^	China	RCT	Those aged 50 years or more with a confirmed diagnosis of cervical Herpes Zoster with unilateral lesions appearing between level C5 and level C7; rash present within 3 days; zoster associated pain	Control: received the standard antiviral treatment (famciclovir 250 mg 3 times daily for 7 days) plus ultrasound-guided CRB with similar-looking placebo. Intervention: received standard antiviral treatment plus ultrasound guided cervical nerve root block (CRB) with mixed drug liquid (lidocaine 4 mg/mL + triamcinolone 1 mg/mL + cobamamide 0.5 mg/mL + normal saline).	Incidence of Post-herpetic neuralgia (PHN) at 12 weeks Control: 23/70 (32.9%); Intervention: 13/70 (18.6%) No serious adverse events were noted in any of the two groups
Van Wijck AJM et al (2006)^15^	Netherlands	RCT	Herpes zoster within 7 days after onset of the rash, dermatome below C6, age older than 50 years	Control: Patients randomised to the control group received the current standard treatment for herpes, consisting of analgesics as needed and antiviral edication Intervention: Patients allocated to the nerve block group also received the standard treatment. Additionally, they received a mixture of 80 mg methylprednisolone acetate and 10 mg bupivacaine (0·25% weight/volume) injected epidurally.	Incidence of Post-herpetic neuralgia (PHN) at 12 weeks Control: 63/266 (24.0%); Intervention: 58/275 (21.0%) No serious adverse events were noted in any of the two groups
Ji G et al (2009)^16^	China	RCT	Herpes zoster within 7 days after onset of the rash, dermatome below C6, age older than 50 yrs.	Control: Patients randomized to the control group received the current standard treatment for herpes (oral administration of 800 mg acyclovir, 5 times daily for 7 days, and analgesics as needed). Intervention: Patients allocated to this group also received standard treatment. Additionally, they were given repetitive nerve block with a mixture of 10 mL 0.25% bupivacaine and 40 mg methylprednisolone acetate every 48 h for a week (total 4 injections at each desired level)	Incidence of Post-herpetic neuralgia (PHN) at 12 weeks Control: 18/60 (30.0%); Intervention: 4/57 (7.0%) Mean (SD) Visual Analogue Scale (VAS) score at 12 weeks Control (N=18): 5.45 (1.25); Intervention (N=4): 5.35 (1.30)
Hwang SM et al (1999)^17^	South Korea	Prospective non-randomized comparative clinical trial	Patients with Herpes Zoster within 14 days of onset of disease; Mean age of around 60 years	Control: Intravenous acyclovir (5 mg/kg three times a day for 7 days) Intervention: Intravenous acyclovir with nerve block (methylprednisolone acetate and 0.125% bupivacaine) for 7 days	Mean (SD) duration of pain (in days) Control: 31.6 (17.6); Intervention: 18.5 (9.3) No serious adverse events were noted in any of the two groups
Makharita MY et al (2012)^18^	Egypt	RCT	Adult patients aged over 50 years with herpetic eruption of less than 2 weeks and under or received appropriate antiviral therapy	Control: stellate ganglion block using 8 mL saline as placebo Intervention: stellate ganglion block using 6 mL bupivacaine 0.125% plus 8 mg dexamethasone in a total volume of 8 mL	Incidence of Post-herpetic neuralgia (PHN) at 12 weeks Control: 8/30 (26.7%); Intervention: 2/31 (6.5%) Mean (SD) duration of pain (in days) Control: 43.6 (28.7); Intervention: 23.8 (18.0) Mean (SD) VAS score at 12 weeks Control: 1.1 (1.8); Intervention: 0.13 (0.5) Adverse effects Drowsiness Control: 20/30 (66.7%); Intervention: 17/31 (54.8%)
Lee YB et al (1999)^19^	South Korea	RCT	HZ patients located in the cervical through the sacral dermatome within 20 days of onset of the disease	Control: Standard therapy with antiviral agents (acyclovir 5 mg/kg 3 times a day for 5−7 days) and supplementary analgesics Intervention: Standard therapy plus repetitive nerve block (bupivacaine with methylprednisolone)	Incidence of Post-herpetic neuralgia (PHN) at 12 weeks Control: 1/35 (2.85%); Intervention: 2/40 (5.0%)

Additionally, the pooled risk ratios (RR) suggest a significant beneficial effect of a combination of local anaesthetic and steroid injection in reducing the incidence of post-herpetic neuralgia, compared to standard care/placebo. Compared to standard care/placebo, those receiving a combination of local anaesthetic and steroid injection had 55% lower risk of PHN at three months from onset of rash (RR 0.45; 95% CI, 0.29; 0.70; I^2^=55.8%) (Fig.2). There was no evidence of publication bias (P-value=0.089).

**Fig.3:**
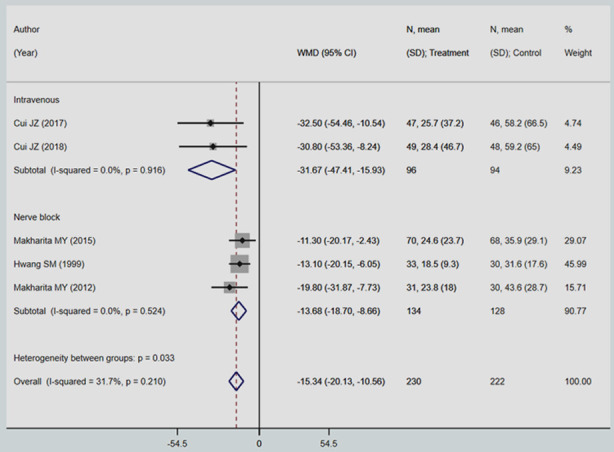
Pooled effect of local anaesthetic and steroid injection on duration of neuralgic pain (in days).

The maximum reduction in the duration of pain (in days) was noted in subjects that received the intervention intravenously (WMD -31.67; 95%CI, -47.41, -15.93; I^2^=0.0%) followed by nerve block (WMD -13.68; 95%CI, -18.70, -8.66; I^2^=0.0%) (Fig.3). There were no studies on subcutaneous mode of delivery of local anaesthetic and steroid that reported this outcome.

From the overall pooling of the studies, irrespective of the mode of delivery of the intervention, there were significant differences in the duration of neuralgic pain (in days) between subjects receiving a combination of local anaesthetic and steroid injection and those that received standard care/placebo. The overall mean difference (in days) between the two groups was statistically significant (WMD -15.34; 95%CI, -20.13, -10.56; I^2^=31.7%) (Fig.3). Among those receiving the intervention, the mean duration of pain was around 15 days lesser than those that received standard care/placebo. There was no evidence of publication bias (P-value=0.153).

**Supplementary Fig.1:**
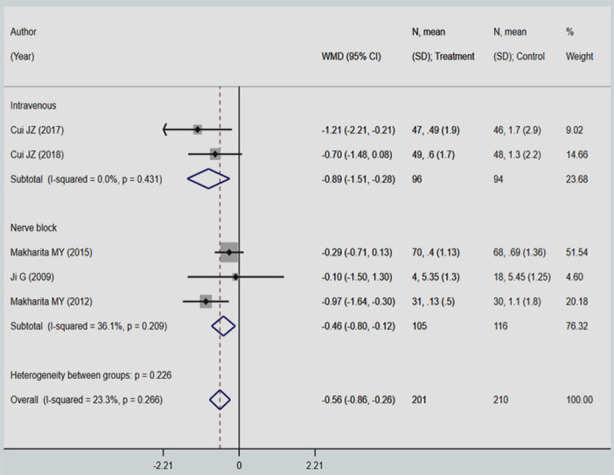
Pooled effect of local anaesthetic and steroid injection on mean scores on Visual Analogue Scale.

Among those receiving local anaesthetic and steroid through intravenous route, the intensity of neuralgic pain was significantly lesser compared to those that received standard care/placebo (WMD -0.89; 95% CI, -1.51, -0.28; I^2^=0.0%) (Supplementary Fig.1). In those receiving nerve block, there was also a significant reduction in the intensity of pain, although lesser than intravenous route ((WMD -0.46; 95% CI, -0.80, -0.12; I^2^=36.1%). There were no studies on subcutaneous mode of delivery of local anaesthetic and steroid that reported this outcome.

In the overall pooled analyses, among those receiving the intervention, the intensity of neuralgic pain was lesser than those that received standard care/placebo at 3 months from the onset of rash. The mean difference between the two groups was statistically significant (WMD -0.56; 95%CI, -0.86, -0.26; I^2^=23.3%) (Supplementary Fig.I). There was no evidence of publication bias (P-value=0.438).

### Adverse events

**Supplementary Fig.2:**
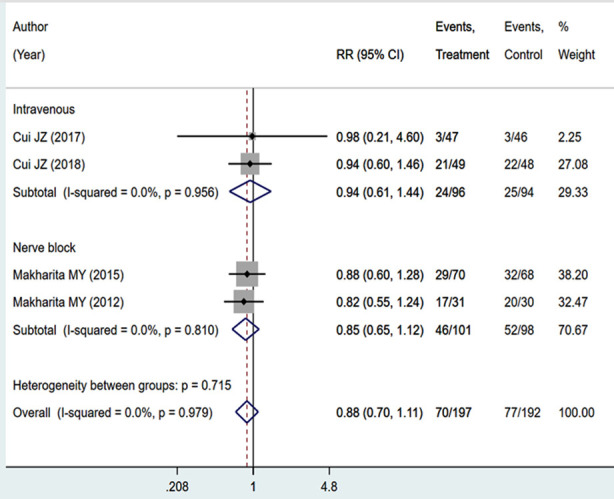
Pooled risk of having drowsiness in those receiving local anaesthetic and steroid injection, compared to those receiving standard medical care and/or placebo.

The included studies did not report an increased incidence of adverse events in those receiving a combination of local anaesthetic and steroid injection, compared to those receiving standard care/placebo. The intervention was not associated with an increased risk of drowsiness (RR 0.88, 95%CI; 0.70; 1.11; I^2^=0.0%) (Supplementary Fig.2). Even with regards to the mode of administration i.e., intravenous (RR 0.94, 95%CI; 0.61; 1.44; I^2^=0.0%) or nerve block (RR 0.85, 95%CI; 0.65; 1.12; I^2^=0.0%), there were no differential risk compared to the standard care (Supplementary Fig.2). There was no evidence of publication bias (P-value=0.602).

The evidence on risk of post-herpetic neuralgia and on the duration of neuralgic pain was assessed to be of “low quality” whereas for the visual analogue scores and risk of drowsiness (denoting adverse events), the quality was “very low” (Table-I).

**Table I:** Quality of evidence for according to the GRADE criteria.

Outcomes	Number of studies (Design); No. of participants	Effect size (95% CI)	Characteristics of included studies					

			Risk of bias	Inconsistency	Indirectness ^a^	Imprecision	Publication bias	Overall GRADE quality score
Incidence of post-herpetic neuralgia	9 (RCTs); n= 1362	RR 0.45 (0.29, 0.70)	Not serious	Not serious	Serious	Not serious	Undetected	⊕⊕○○LOW
Duration of neuralgic pain	5 (4 RCTs; 1 non-RCT); n=452	WMD -15.34 (-20.13, -10.56)	Serious ^b^	Not serious	Serious	Not Serious	Undetected	⊕⊕○○ LOW
Mean Visual Analogue Score	5 (RCTs); n= 411	WMD -0.56 (-0.86, -0.26)	Not serious	Not serious	Serious	Serious^c^	Undetected	⊕○○○ VERY LOW
Risk of drowsiness (Adverse event)	4 (RCTs); n=389	RR 0.88 (0.70, 1.11)	Not serious	Not serious	Serious	Serious^d^	Undetected	⊕○○○ VERY LOW

a Studies were done in different geographic settings. Further, studies used different concentrations of local anaesthetic and steroid and there were differences in the mode of administration i.e. intravenous, subcutaneous and neural blocks;

b Due to inclusion of one non-randomized trial, the score has been downgraded;

c Criteria for optimal information size (OIS) was not met;

d Criteria for optimal information size (OIS) was not met and the 95% CI overlaps no effect and includes important benefit and harm.

## DISCUSSION

Our current understanding of the PHN is that herpes zoster infection initiated an acute inflammatory state in the dorsal root ganglion which subsequently leads to increased sympathetic vasoconstriction and consequent neural damage.^20^,^21^ The beneficial effects of injecting local anaesthetic and steroids could be because of sympathetic blockage, thereby inhibiting vasoconstriction and due to anaesthetization of the dorsal root ganglion and posterior spinal nerve.^20^–^22^ Numerous pharmacotherapeutic agents have been used to treat or prevent PHN. These agents include antiviral medications, analgesics, corticosteroids and in some cases, antidepressants and anticonvulsants.^23^ While the use of antiviral medications has been shown to reduce the acute herpes zoster pain and accelerates healing of the lesions, it’s benefits in prevention of PHN remain non-conclusive.^23^ Oral corticosteroid in combination with an antiviral therapy alleviates pain from herpes zoster (HZ) but minimal effect was noted in terms of reducing the severity and duration of the pain.^23^ Use of analgesics remains controversial as it can reduce pain but on the other hand, can also aggravate the lesions of HZ.^23^ Use of opioids is associated with side effects such as nausea, dizziness, somnolence, and in some case, constipation.^23^ The current evidence of the efficacy of using combined local anaesthetic and steroids in the prevention and management of PHN is promising but not well established. In this review, we aimed to synthesize all the available randomized controlled trials and present the most updated evidence.

The current meta-analysis was undertaken to provide an updated synthesized estimate of the efficacy of local anaesthetics in combination with injectable steroids in prevention and management of post-herpetic neuralgia and in reducing the duration of neuralgic pain, compared to the standard care and/or placebo. We found that use of a combination of local anaesthetic and injectable steroids reduced the risk of experiencing PHN by 55% and the duration of pain. We further observed no significant increase in the adverse side effects with use of this combination. Our findings corroborate with the recently conducted review by Kim HJ et al.^6^ that showed that nerve block using local anaesthetics and/or steroid injections reduced the incidence of post-herpetic neuralgia by 57% and reduced the duration of zoster related pain, in comparison to the standard of care. We also noted that there were differential effects on incidence of PHN, duration of neuralgic pain and mean VAS score based on whether the intervention was delivered intravenously or through nerve block. Use of any of these modes should be governed by treating clinician’s or the patient’s preference as well as the skills of the clinician. The overall quality of evidence generated through the pooled analysis was “low to very low” and therefore, calls for more comprehensive and methodologically robust randomized controlled trials on this important clinical issue.

### Limitations of the study

Firstly, for some of the studies, the sample size was small and could have affected the overall pooled estimates as well as limited the generalizability of the evidence. Secondly, the present meta-analysis cannot provide clear guidelines regarding frequency, duration, or type of local anesthetics and steroids to be used for the prevention and management of PHN.

## CONCLUSION

The meta-analysis provides some evidence to support the use of combined local anaesthetic and steroids in reducing risk of post-herpetic neuralgia and duration of neuralgic pain in patients with herpes zoster rash. Further, the use of this combination is not associated with increase in adverse events. Overall, the GRADE assessments indicate that the current evidence is not of high quality and more robust and methodologically sound clinical trials on this aspect needs to be done.

### Authors’ contributions:

**XZ:** Conceived and designed the study.

**ZW and YX:** Collected the data and performed the analysis.

**XZ:** Involved in the Writing of the manuscript and is responsible for integrity of the study.

**YX:** Edited the manuscript.

All authors have read and approved the final manuscript.
